# Incorporating medical interventions into carrier probability estimation for genetic counseling

**DOI:** 10.1186/1471-2350-8-13

**Published:** 2007-03-22

**Authors:** Hormuzd A Katki

**Affiliations:** 1 Division of Cancer Epidemiology and Genetics, National Cancer Institute, NIH, DHHS, 6120 Executive Blvd. Room 8044 Rockville, MD 20852, USA

## Abstract

**Background:**

Mendelian models for predicting who may carry an inherited deleterious mutation of known disease genes based on family history are used in a variety of clinical and research activities. People presenting for genetic counseling are increasingly reporting risk-reducing medical interventions in their family histories because, recently, a slew of prophylactic interventions have become available for certain diseases. For example, oophorectomy reduces risk of breast and ovarian cancers, and is now increasingly being offered to women with family histories of breast and ovarian cancer. Mendelian models should account for medical interventions because interventions modify mutation penetrances and thus affect the carrier probability estimate.

**Methods:**

We extend Mendelian models to account for medical interventions by accounting for post-intervention disease history through an extra factor that can be estimated from published studies of the effects of interventions. We apply our methods to incorporate oophorectomy into the BRCAPRO model, which predicts a woman's risk of carrying mutations in *BRCA1 *and *BRCA2 *based on her family history of breast and ovarian cancer. This new BRCAPRO is available for clinical use.

**Results:**

We show that accounting for interventions undergone by family members can seriously affect the mutation carrier probability estimate, especially if the family member has lived many years post-intervention. We show that interventions have more impact on the carrier probability as the benefits of intervention differ more between carriers and non-carriers.

**Conclusion:**

These findings imply that carrier probability estimates that do not account for medical interventions may be seriously misleading and could affect a clinician's recommendation about offering genetic testing. The BayesMendel software, which allows one to implement any Mendelian carrier probability model, has been extended to allow medical interventions, so future Mendelian models can easily account for interventions.

## Background

People who are concerned that their family has a high prevalence of disease may seek counseling to assess their probability of carrying inherited genetic mutations that cause disease [1]. The carrier probability is a crucial component in a person's decision to take a genetic test, to undergo frequent disease screening, or to consider prophylactic medical interventions.

To aid such people ("consultands"), genetic counselors use statistical models that predict whether the consultand carries deleterious mutations by using the consultand's reported family history of disease. Mendelian models use Mendel's laws and Bayes's rule to combine family history information with each mutation's known prevalence and penetrance to determine the probability that the consultand is a mutation carrier [2]. For syndromes whose onset is apparent early in life and where the mutations have complete penetrance, the carrier probability from a Mendelian model can be computed from simple mathematical formulae or tables of risks [1]. But for complex syndromes, the Mendelian model must account for many more factors, such as age-dependent incomplete penetrances, potential censoring of disease whose onset may not occur over a lifetime, and environmental determinants [3].

For complex syndromes, computing the carrier probability from a Mendelian model requires software. The trouble of using software is often worthwhile because simple risk tables often do not provide as accurate a carrier probability estimate as computing the carrier probability from a Mendelian model [4]. The freely-available software package BayesMendel [5] allows anyone to implement a Mendelian model and is the computational engine behind BRCAPRO [6,7] and MMRPRO [8]. BRCAPRO estimates the probability that a consultand carries a deleterious mutation in the *BRCA1 *[MIM 113705] and *BRCA2 *[MIM 600185] genes, based on family history of breast and ovarian cancer, while MMRPRO computes the probability of carrying a mutation in the DNA mismatch repair genes *MLH1 *[MIM 120436], *MSH2 *[MIM 609309], and *MSH6 *[MIM 600678] given family history of colorectal and endometrial cancer. Genetic counselors use BRCAPRO and MMRPRO via CancerGene [9], which provides a user-friendly graphical interface. BRCAPRO has a proven clinical track-record [4] and will be the example Mendelian model in this paper. We have incorporated the extensions we describe in this paper into BayesMendel and BRCAPRO, and these are available for use by genetic counselors.

Mendelian models can be extended as knowledge accrues about complex disease genetics, translating cutting-edge genetic research into use for genetic counselors [3]. In this paper, we extend Mendelian models to account for medical interventions undergone by family members. For example, a woman at high risk of breast or ovarian cancer may undergo bilateral salpingo-oophorectomy ("oophorectomy"), the removal of the ovaries and fallopian tubes. Although oophorectomy has adverse consequences for other diseases and for quality of life, oophorectomy halves risk of breast cancer and eliminates risk of ovarian cancer [10]. However, peritoneal cancer near the gynecologic tract (which is often indistinguishable from ovarian cancer) can still occur [11].

Furthermore, a panoply of prophylactic interventions are now available for familial cancer syndromes, and consultands are increasingly reporting family members who have undergone interventions. For example, oophorectomy is increasingly exercised by women at high risk of breast and ovarian cancers, especially *BRCA *mutation carriers. Most studies show that about 50% of *BRCA *carriers undergo oophorectomy [12]. Oophorectomy is so commonly chosen because it can prevent both breast and ovarian cancer. Women concerned only about breast cancer risk may undergo prophylactic tamoxifen or bilateral mastectomy; women have attempted to reduce their ovarian cancer mortality with frequent CA-125 tests or transvaginal ultrasounds [12]. Prophylactic interventions are also available for people at high risk of familial colon and endometrial cancer, especially those with mutations in DNA mismatch repair genes. Such individuals may undergo colectomy [13] or hysterectomy with oophorectomy [14], and these interventions have implications for MMRPRO.

Medical interventions must be accounted for by Mendelian models for two reasons. First, the act of a relative choosing to undergo intervention may imply that that relative knows that she is at high risk and could be a carrier, unbeknownst to the consultand. Second, interventions alter mutation penetrances. Oophorectomy reduces risk, in carriers, of breast cancer by 54% [15] and of ovarian or peritoneal cancer from 80% [11] to 96% [16]. Ignoring interventions means that the model untenably assumes that family members who have undergone intervention have the same penetrances as those who have not.

**Figure 1:**
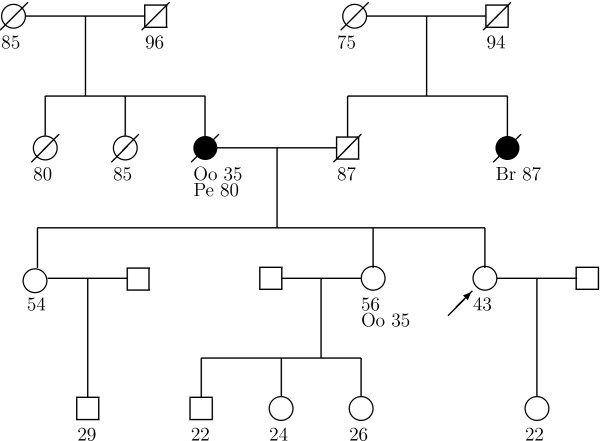
**Example Family Tree**. Family tree with breast (Br) and peritoneal (Pe) cancer history, and oophorectomy (Oo). The arrow points to the consultand. Circles are females, squares are males. Slash means the relative died, dark shape means the relative developed cancer, light shape and no slash means the relative is alive with no cancer, and the age of those outcomes is below each member.

Currently, except for BRCAPRO, other mutation prediction models ignore the effects of medical interventions taken by family members. Ignoring oophorectomy can seriously affect the *BRCA *carrier probability estimate, as for the family in figure 1. In this family, a sister has never developed cancer, but she underwent oophorectomy at a young age. Also, the mother's peritoneal cancer after oophorectomy is at a very old age, as is the aunt's cancer. Cancer at old ages is usually weak evidence of a *BRCA *mutation, and ignoring the oophorectomies, the consultand's BRCAPRO carrier probability estimate is only 3%. However, the mother lived more of her life without ovaries, so accounting for her oophorectomy is important. Getting cancer after oophorectomy is more evidence for a mutation and when BRCAPRO accounts for oophorectomy (as detailed in the Methods), the carrier probability jumps to 13%. This difference is especially critical because many counselors offer genetic testing to the consultand once the probability hits 10% [17] and health insurers may not pay for the test unless the probability is high enough [18].

Table 1 shows how the BRCAPRO carrier probability changes when accounting for oophorectomy for different family histories based on figure 1. Table 1 shows that accounting for the sister's oophorectomy is not as important as the mother's oophorectomy. This is because the mother developed cancer after oophorectomy. In particular, we estimate that while carriers have a reduction of 88% in the risk of ovarian or peritoneal cancer after oophorectomy (see Methods), non-carriers enjoy a 95% risk reduction [19]. Thus getting peritoneal cancer after oophorectomy is additional evidence for being a carrier. Also in table 1, if the sister develops breast cancer at 56, accounting for oophorectomies changes the carrier probabilities from 11% to 29%, which might affect the decision to offer testing since 11% is close to the 10% guideline. If the sister develops peritoneal cancer, the probability changes from 25% to 44%, a big change, but genetic testing would probably be offered in either case. If the mother instead had breast cancer at 80, the carrier probability only goes from 1% to 1.4%, but is an increase of 40%. Since interventions multiplicatively affect the carrier probability (detailed in Methods), percent change is a noteworthy metric.

**Table 1:** Effect on BRCAPRO Carrier Probability of Ignoring vs. Including Oophorectomy for Families Based on Figure 1.

Family	Ignoring all Oophorectomies	Including all Oophorectomies
As in Figure 1	3%	13%
No oophorectomy for sister	3%	12%
No oophorectomy for mother	3%	4%
Sister has breast cancer at 56	11%	29%
Sister has peritoneal cancer at 56	25%	44%
Mother has only breast cancer at 80	1%	1.4%

In this paper, we show how to incorporate medical interventions into Mendelian models. The only extra quantity needed is a post-intervention factor for those who chose intervention. This post-intervention factor can be estimated using the reduction in disease hazard caused by the intervention, which is commonly estimated in studies of the effects of interventions. We show that, as long as the consultand accurately reports family history and is aware of the genetic test results on any relatives, then we do not need to model the effect of family history and carrier status on choosing to (or not to) undergo oophorectomy. We detail how we chose to incorporate oophorectomy into BRCAPRO. Given current uncertainties about the effects of oophorectomy, we show the impact of oophorectomy for different instructive scenarios. These scenarios show that the importance of accounting for interventions increases as the benefits of intervention differ more between carriers and non-carriers. We have incorporated interventions into BayesMendel, allowing anyone to incorporate any intervention into any Mendelian model. In particular, oophorectomy has been incorporated into BRCAPRO and this new BRCAPRO has been released to counselors for clinical use.

## Methods

### Computing the Carrier Probability

Mendelian models require knowledge of which disease each relative developed and the age when it was diagnosed. For example, for BRCAPRO, the diseases are age at ovarian cancer or breast cancer onset. Although there can be many causes of censoring [20], we restrict to a single independent non-informative censoring being the minimum of the age alive after which no information is known or the age of death. In this framework, everyone is eventually censored but disease history up to that age of censoring is observed. Denote the age at which censoring occurs for each family member *i *(*i *= 0 is the consultand) as *U*_*i*_.

A Mendelian model considers *D *types of diseases that could occur. Each person has a binary vector indicating disease history *c*_*i *_= (*c*_*i*,1_, ..., *c*_*i*,*D*_) where *c*_*i*,*k *_= 1 indicates that disease *k *occurred at age *y*_*i*,*k *_and let *y*_*i *_= (*y*_*i*,1_, ..., *y*_*i,D*_) be the vector of all ages of disease occurrence. In *y*_*i*_, the age for any disease that did not occur is irrelevant, and so can be set to 0. Denoting disease information as *T*_*i *_= {*y*_*i*_, *c*_*i*_}, each person's history is the information *H*_*i *_= {*U*_*i*_, *T*_*i*_} and the full family history is the collection *H *= {*H*_0_, *H*_1_, ...}.

Additionally, each person can have auxiliary variables *x*_*i *_and let *x *= {*x*_0_, *x*_1_, ...}. Auxiliaries can be any extra information known by the consultand, for example, environmental factors, genetic test results, or ethnicity. For example, in BRCAPRO, *x*_0 _indicates if the consultand is of Ashkenazi Jewish ancestry, an ethnic group with increased prevalence of *BRCA *mutations. Implicitly, all probabilities in this paper will condition on *x*, so for simplicity we only explicitly show *x *in the conditioning when useful.

Mendelian models assume that individuals independently inherit one allele from each parent at each autosomal locus and that the alleles are either normal or mutated. Let *γ*_*i *_= 0,1 indicate carrying the genotype(s) that confer(s) disease risk: for example, *γ*_*i *_= 1 for a dominant trait when the member carries at least 1 mutant allele, but for a recessive trait *γ*_*i *_= 1 implies that the relative carries two mutant alleles. We call *γ*_*i *_the carrier status. The prevalence of *γ*_*i *_= 1 amongst people with consultand-specific auxiliaries *x*_0 _is *π*_*x*_.

The aim of a Mendelian model is to compute the consultand's carrier probability *P*(*γ*_0 _= 1|*H*, *x*). By Bayes rule, the odds of the consultand being a carrier is a product of the carrier odds in the population and the Bayes Factor (BF):

P(γ0=1|H,x)P(γ0=0|H,x)=πx1−πx×BF(H), whereBF(H)=P(H|γ0=1,x)P(H|γ0=0,x).     (1)
 MathType@MTEF@5@5@+=feaafiart1ev1aaatCvAUfKttLearuWrP9MDH5MBPbIqV92AaeXatLxBI9gBaebbnrfifHhDYfgasaacH8akY=wiFfYdH8Gipec8Eeeu0xXdbba9frFj0=OqFfea0dXdd9vqai=hGuQ8kuc9pgc9s8qqaq=dirpe0xb9q8qiLsFr0=vr0=vr0dc8meaabaqaciaacaGaaeqabaqabeGadaaakeaadaWcaaqaaiabdcfaqjabcIcaOGGaciab=n7aNnaaBaaaleaacqaIWaamaeqaaOGaeyypa0JaeGymaeJaeiiFaWNaemisaGKaeiilaWIaemiEaGNaeiykaKcabaGaemiuaaLaeiikaGIae83SdC2aaSbaaSqaaiabicdaWaqabaGccqGH9aqpcqaIWaamcqGG8baFcqWGibascqGGSaalcqWG4baEcqGGPaqkaaGaeyypa0ZaaSaaaeaacqWFapaCdaWgaaWcbaGaemiEaGhabeaaaOqaaiabigdaXiabgkHiTiab=b8aWnaaBaaaleaacqWG4baEaeqaaaaakiabgEna0kabdkeacjabdAeagjabcIcaOiabdIeaijabcMcaPiabcYcaSiabbccaGGqaaiab+Dha3jab+HgaOjab+vgaLjab+jhaYjab+vgaLjab+bcaGiab+bcaGiabdkeacjabdAeagjabcIcaOiabdIeaijabcMcaPiabg2da9maalaaabaGaemiuaaLaeiikaGIaemisaGKaeiiFaWNae83SdC2aaSbaaSqaaiabicdaWaqabaGccqGH9aqpcqaIXaqmcqGGSaalcqWG4baEcqGGPaqkaeaacqWGqbaucqGGOaakcqWGibascqGG8baFcqWFZoWzdaWgaaWcbaGaeGimaadabeaakiabg2da9iabicdaWiabcYcaSiabdIha4jabcMcaPaaacqGGUaGlcaWLjaGaaCzcamaabmaabaGaeGymaedacaGLOaGaayzkaaaaaa@8453@

The BF is a ratio of likelihoods. We compute the likelihood, assuming that each member's phenotype *H*_*i *_is independent of all other members' phenotypes *H*_-*i *_and auxiliaries *x*_-*i *_given that member's carrier status *γ*_*i *_and auxiliary variables *x*_*i *_[21]:

*P*(*H*_*i*_|*γ*_*i*_, *x*, *H*_-*i*_) = *P*(*H*_*i*_|*γ*_*i*_, *x*_*i*_).     (2)

The likelihood is

P(H|γ0,x)=P(H0|γ0,x0)∑γ1,...γn=01P(H1,...Hn|γ0,...γn,x)P(γ1,...γn|γ0,x)=P(H0|γ0,x0)∑γ1,...γn=01(∏i=1nP(Hi|γi,xi))P(γ1,...γn|γ0,x).     (3)
 MathType@MTEF@5@5@+=feaafiart1ev1aaatCvAUfKttLearuWrP9MDH5MBPbIqV92AaeXatLxBI9gBaebbnrfifHhDYfgasaacH8akY=wiFfYdH8Gipec8Eeeu0xXdbba9frFj0=OqFfea0dXdd9vqai=hGuQ8kuc9pgc9s8qqaq=dirpe0xb9q8qiLsFr0=vr0=vr0dc8meaabaqaciaacaGaaeqabaqabeGadaaakeaafaqaaeGadaaabaGaemiuaaLaeiikaGIaemisaGKaeiiFaWhcciGae83SdC2aaSbaaSqaaiabicdaWaqabaGccqGGSaalcqWG4baEcqGGPaqkaeaacqGH9aqpaeaacqWGqbaucqGGOaakcqWGibasdaWgaaWcbaGaeGimaadabeaakiabcYha8jab=n7aNnaaBaaaleaacqaIWaamaeqaaOGaeiilaWIaemiEaG3aaSbaaSqaaiabicdaWaqabaGccqGGPaqkdaaeWbqaaiabdcfaqjabcIcaOiabdIeainaaBaaaleaacqaIXaqmaeqaaOGaeiilaWIaeiOla4IaeiOla4IaeiOla4IaemisaG0aaSbaaSqaaiabd6gaUbqabaGccqGG8baFcqWFZoWzdaWgaaWcbaGaeGimaadabeaakiabcYcaSiabc6caUiabc6caUiabc6caUiab=n7aNnaaBaaaleaacqWGUbGBaeqaaOGaeiilaWIaemiEaGNaeiykaKIaemiuaaLaeiikaGIae83SdC2aaSbaaSqaaiabigdaXaqabaGccqGGSaalcqGGUaGlcqGGUaGlcqGGUaGlcqWFZoWzdaWgaaWcbaGaemOBa4gabeaakiabcYha8jab=n7aNnaaBaaaleaacqaIWaamaeqaaOGaeiilaWIaemiEaGNaeiykaKcaleaacqWFZoWzdaWgaaadbaGaeGymaedabeaaliabcYcaSiabc6caUiabc6caUiabc6caUiab=n7aNnaaBaaameaacqWGUbGBaeqaaSGaeyypa0JaeGimaadabaGaeGymaedaniabggHiLdaakeaaaeaacqGH9aqpaeaacqWGqbaucqGGOaakcqWGibasdaWgaaWcbaGaeGimaadabeaakiabcYha8jab=n7aNnaaBaaaleaacqaIWaamaeqaaOGaeiilaWIaemiEaG3aaSbaaSqaaiabicdaWaqabaGccqGGPaqkdaaeWbqaamaabmaabaWaaebCaeaacqWGqbaucqGGOaakcqWGibasdaWgaaWcbaGaemyAaKgabeaakiabcYha8jab=n7aNnaaBaaaleaacqWGPbqAaeqaaOGaeiilaWIaemiEaG3aaSbaaSqaaiabdMgaPbqabaGccqGGPaqkaSqaaiabdMgaPjabg2da9iabigdaXaqaaiabd6gaUbqdcqGHpis1aaGccaGLOaGaayzkaaGaemiuaaLaeiikaGIae83SdC2aaSbaaSqaaiabigdaXaqabaGccqGGSaalcqGGUaGlcqGGUaGlcqGGUaGlcqWFZoWzdaWgaaWcbaGaemOBa4gabeaakiabcYha8jab=n7aNnaaBaaaleaacqaIWaamaeqaaOGaeiilaWIaemiEaGNaeiykaKIaeiOla4caleaacqWFZoWzdaWgaaadbaGaeGymaedabeaaliabcYcaSiabc6caUiabc6caUiabc6caUiab=n7aNnaaBaaameaacqWGUbGBaeqaaSGaeyypa0JaeGimaadabaGaeGymaedaniabggHiLdaaaOGaaCzcaiaaxMaadaqadaqaaiabiodaZaGaayjkaiaawMcaaaaa@CAC2@

This depends on family history only through the contributions *P*(*H*_*i*_|*γ*_*i*_, *x*_*i*_), so we focus on computing these. To ease notation, auxiliaries *x*_*i *_are always implicitly conditioned on, and will be made explicit only when useful. For more details, a discussion of underlying assumptions, and an explicit derivation of the Bayes Factor [5,21].

Each person's likelihood contribution *P*(*H*_*i*_|*γ*_*i*_, *x*_*i*_) will be computed assuming that competing risks are independent given carrier status and auxiliaries [20]. This is plausible for BRCAPRO because time to ovarian cancer and ipsi/contra-lateral breast cancers appear to be mutually independent in *BRCA *mutation carriers, except for dependence caused by medical interventions like oophorectomy [22,23] and interventions are explicitly handled in this paper. Auxiliaries *x*_*i *_can include all information necessary to make the assumption more plausible [20]. Thus for simplicity and relevance to BRCAPRO, we restrict to independent competing risks.

Under independent competing risks, define the hazard for each disease *k *> 0 at age *T *given carrier status as *λ*_*k*_(*y*|*γ*). The disease-specific survival, the probability of surviving disease *k *to age *y*, is

Sk(y|γ)=exp{−∫0yλk(u|γ) du}.     (4)
 MathType@MTEF@5@5@+=feaafiart1ev1aaatCvAUfKttLearuWrP9MDH5MBPbIqV92AaeXatLxBI9gBaebbnrfifHhDYfgasaacH8akY=wiFfYdH8Gipec8Eeeu0xXdbba9frFj0=OqFfea0dXdd9vqai=hGuQ8kuc9pgc9s8qqaq=dirpe0xb9q8qiLsFr0=vr0=vr0dc8meaabaqaciaacaGaaeqabaqabeGadaaakeaacqWGtbWudaWgaaWcbaGaem4AaSgabeaakiabcIcaOiabdMha5jabcYha8HGaciab=n7aNjabcMcaPiabg2da9Gqaciab+vgaLjab+Hha4jab+bhaWnaacmqabaGaeyOeI0Yaa8qmaeaacqWF7oaBdaWgaaWcbaGaem4AaSgabeaakiabcIcaOiabdwha1jabcYha8jab=n7aNjabcMcaPiabbccaGiabdsgaKjabdwha1bWcbaGaeGimaadabaGaemyEaKhaniabgUIiYdaakiaawUhacaGL9baacqGGUaGlcaWLjaGaaCzcamaabmaabaGaeGinaqdacaGLOaGaayzkaaaaaa@547A@

The disease-specific density, the probability of getting disease *k *at age *y*, is

*f*_*k*_(*y*|*γ*) = *λ*_*k*_(*y*|*γ*) × *S*_*k*_(*y*|*γ*).     (5)

Each likelihood contribution *P*(*H*_*i*_|*γ*_*i*_) is an ignorably right-censored survival likelihood contribution, which is the product of disease-specific densities for diseases that occurred and the disease-specific survivals for diseases that did not occur [20]:

P(Hi|γi)∝∏k=1Dfk(yik|γi)cikSk(ui|γi)1−cik.     (6)
 MathType@MTEF@5@5@+=feaafiart1ev1aaatCvAUfKttLearuWrP9MDH5MBPbIqV92AaeXatLxBI9gBaebbnrfifHhDYfgasaacH8akY=wiFfYdH8Gipec8Eeeu0xXdbba9frFj0=OqFfea0dXdd9vqai=hGuQ8kuc9pgc9s8qqaq=dirpe0xb9q8qiLsFr0=vr0=vr0dc8meaabaqaciaacaGaaeqabaqabeGadaaakeaacqWGqbaucqGGOaakcqWGibasdaWgaaWcbaGaemyAaKgabeaakiabcYha8HGaciab=n7aNnaaBaaaleaacqWGPbqAaeqaaOGaeiykaKIaeyyhIu7aaebCaeaacqWGMbGzdaWgaaWcbaGaem4AaSgabeaakiabcIcaOiabdMha5naaBaaaleaacqWGPbqAcqWGRbWAaeqaaOGaeiiFaWNae83SdC2aaSbaaSqaaiabdMgaPbqabaGccqGGPaqkdaahaaWcbeqaaiabdogaJnaaBaaameaacqWGPbqAcqWGRbWAaeqaaaaakiabdofatnaaBaaaleaacqWGRbWAaeqaaOGaeiikaGIaemyDau3aaSbaaSqaaiabdMgaPbqabaGccqGG8baFcqWFZoWzdaWgaaWcbaGaemyAaKgabeaakiabcMcaPmaaCaaaleqabaGaeGymaeJaeyOeI0Iaem4yam2aaSbaaWqaaiabdMgaPjabdUgaRbqabaaaaaWcbaGaem4AaSMaeyypa0JaeGymaedabaGaemiraqeaniabg+GivdGccqGGUaGlcaWLjaGaaCzcamaabmaabaGaeGOnaydacaGLOaGaayzkaaaaaa@6850@

### Incorporating Medical Interventions

Medical interventions censor the natural time to disease, leaving only the time to disease after intervention. Along with pre-intervention quantities *Y*_*i*_, *C*_*i*_, *U*_*i*_, there is the age of intervention *I*_*i *_and post-intervention quantities: post-intervention disease types ciI
 MathType@MTEF@5@5@+=feaafiart1ev1aaatCvAUfKttLearuWrP9MDH5MBPbIqV92AaeXatLxBI9gBaebbnrfifHhDYfgasaacH8akY=wiFfYdH8Gipec8Eeeu0xXdbba9frFj0=OqFfea0dXdd9vqai=hGuQ8kuc9pgc9s8qqaq=dirpe0xb9q8qiLsFr0=vr0=vr0dc8meaabaqaciaacaGaaeqabaqabeGadaaakeaacqWGJbWydaqhaaWcbaGaemyAaKgabaGaemysaKeaaaaa@309E@, ages of disease yiI
 MathType@MTEF@5@5@+=feaafiart1ev1aaatCvAUfKttLearuWrP9MDH5MBPbIqV92AaeXatLxBI9gBaebbnrfifHhDYfgasaacH8akY=wiFfYdH8Gipec8Eeeu0xXdbba9frFj0=OqFfea0dXdd9vqai=hGuQ8kuc9pgc9s8qqaq=dirpe0xb9q8qiLsFr0=vr0=vr0dc8meaabaqaciaacaGaaeqabaqabeGadaaakeaacqWG5bqEdaqhaaWcbaGaemyAaKgabaGaemysaKeaaaaa@30CA@, and censoring age UiI
 MathType@MTEF@5@5@+=feaafiart1ev1aaatCvAUfKttLearuWrP9MDH5MBPbIqV92AaeXatLxBI9gBaebbnrfifHhDYfgasaacH8akY=wiFfYdH8Gipec8Eeeu0xXdbba9frFj0=OqFfea0dXdd9vqai=hGuQ8kuc9pgc9s8qqaq=dirpe0xb9q8qiLsFr0=vr0=vr0dc8meaabaqaciaacaGaaeqabaqabeGadaaakeaacqWGvbqvdaqhaaWcbaGaemyAaKgabaGaemysaKeaaaaa@3082@. Let TiI
 MathType@MTEF@5@5@+=feaafiart1ev1aaatCvAUfKttLearuWrP9MDH5MBPbIqV92AaeXatLxBI9gBaebbnrfifHhDYfgasaacH8akY=wiFfYdH8Gipec8Eeeu0xXdbba9frFj0=OqFfea0dXdd9vqai=hGuQ8kuc9pgc9s8qqaq=dirpe0xb9q8qiLsFr0=vr0=vr0dc8meaabaqaciaacaGaaeqabaqabeGadaaakeaacqWGubavdaqhaaWcbaGaemyAaKgabaGaemysaKeaaaaa@3080@ = {yiI
 MathType@MTEF@5@5@+=feaafiart1ev1aaatCvAUfKttLearuWrP9MDH5MBPbIqV92AaeXatLxBI9gBaebbnrfifHhDYfgasaacH8akY=wiFfYdH8Gipec8Eeeu0xXdbba9frFj0=OqFfea0dXdd9vqai=hGuQ8kuc9pgc9s8qqaq=dirpe0xb9q8qiLsFr0=vr0=vr0dc8meaabaqaciaacaGaaeqabaqabeGadaaakeaacqWG5bqEdaqhaaWcbaGaemyAaKgabaGaemysaKeaaaaa@30CA@, ciI
 MathType@MTEF@5@5@+=feaafiart1ev1aaatCvAUfKttLearuWrP9MDH5MBPbIqV92AaeXatLxBI9gBaebbnrfifHhDYfgasaacH8akY=wiFfYdH8Gipec8Eeeu0xXdbba9frFj0=OqFfea0dXdd9vqai=hGuQ8kuc9pgc9s8qqaq=dirpe0xb9q8qiLsFr0=vr0=vr0dc8meaabaqaciaacaGaaeqabaqabeGadaaakeaacqWGJbWydaqhaaWcbaGaemyAaKgabaGaemysaKeaaaaa@309E@} and the post-intervention history be HiI
 MathType@MTEF@5@5@+=feaafiart1ev1aaatCvAUfKttLearuWrP9MDH5MBPbIqV92AaeXatLxBI9gBaebbnrfifHhDYfgasaacH8akY=wiFfYdH8Gipec8Eeeu0xXdbba9frFj0=OqFfea0dXdd9vqai=hGuQ8kuc9pgc9s8qqaq=dirpe0xb9q8qiLsFr0=vr0=vr0dc8meaabaqaciaacaGaaeqabaqabeGadaaakeaacqWGibasdaqhaaWcbaGaemyAaKgabaGaemysaKeaaaaa@3068@ = {UiI
 MathType@MTEF@5@5@+=feaafiart1ev1aaatCvAUfKttLearuWrP9MDH5MBPbIqV92AaeXatLxBI9gBaebbnrfifHhDYfgasaacH8akY=wiFfYdH8Gipec8Eeeu0xXdbba9frFj0=OqFfea0dXdd9vqai=hGuQ8kuc9pgc9s8qqaq=dirpe0xb9q8qiLsFr0=vr0=vr0dc8meaabaqaciaacaGaaeqabaqabeGadaaakeaacqWGvbqvdaqhaaWcbaGaemyAaKgabaGaemysaKeaaaaa@3082@, TiI
 MathType@MTEF@5@5@+=feaafiart1ev1aaatCvAUfKttLearuWrP9MDH5MBPbIqV92AaeXatLxBI9gBaebbnrfifHhDYfgasaacH8akY=wiFfYdH8Gipec8Eeeu0xXdbba9frFj0=OqFfea0dXdd9vqai=hGuQ8kuc9pgc9s8qqaq=dirpe0xb9q8qiLsFr0=vr0=vr0dc8meaabaqaciaacaGaaeqabaqabeGadaaakeaacqWGubavdaqhaaWcbaGaemyAaKgabaGaemysaKeaaaaa@3080@}. If intervention occurs, then set *U*_*i *_= *I*_*i*_. Furthermore, any genetic test results known on relatives can be included as an auxiliary *x*_*test*_. Genetic test results provide important information, and Mendelian models can account for imperfect test sensitivity and specificity [5,24].

**Figure 2:**
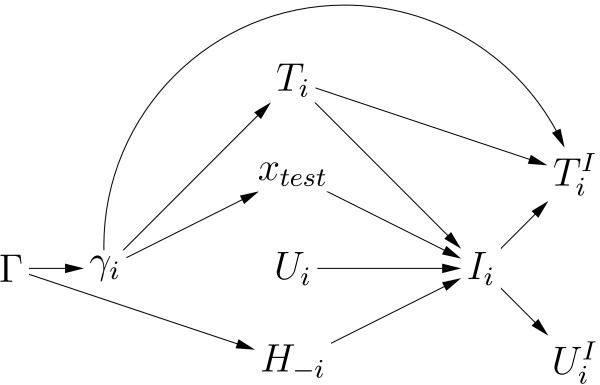
**Relationships amongst pre/post-intervention quantities**. Graph showing conditional dependencies of pre/post-intervention quantities. Γ contains the carrier status of each family member *γ*_*i*_. The factors affecting each member's choice of age of intervention *I*_*i *_are pre-intervention disease history *Y*_*i*_, *C*_*i*_, genetic test results *x*_*test*_, pre-intervention censoring age *U*_*i*_, and everyone else's family history *H*_-*i*_. Post-intervention disease history is YiI
 MathType@MTEF@5@5@+=feaafiart1ev1aaatCvAUfKttLearuWrP9MDH5MBPbIqV92AaeXatLxBI9gBaebbnrfifHhDYfgasaacH8akY=wiFfYdH8Gipec8Eeeu0xXdbba9frFj0=OqFfea0dXdd9vqai=hGuQ8kuc9pgc9s8qqaq=dirpe0xb9q8qiLsFr0=vr0=vr0dc8meaabaqaciaacaGaaeqabaqabeGadaaakeaacqWGzbqwdaqhaaWcbaGaemyAaKgabaGaemysaKeaaaaa@308A@, CiI
 MathType@MTEF@5@5@+=feaafiart1ev1aaatCvAUfKttLearuWrP9MDH5MBPbIqV92AaeXatLxBI9gBaebbnrfifHhDYfgasaacH8akY=wiFfYdH8Gipec8Eeeu0xXdbba9frFj0=OqFfea0dXdd9vqai=hGuQ8kuc9pgc9s8qqaq=dirpe0xb9q8qiLsFr0=vr0=vr0dc8meaabaqaciaacaGaaeqabaqabeGadaaakeaacqWGdbWqdaqhaaWcbaGaemyAaKgabaGaemysaKeaaaaa@305E@ and age last known alive UiI
 MathType@MTEF@5@5@+=feaafiart1ev1aaatCvAUfKttLearuWrP9MDH5MBPbIqV92AaeXatLxBI9gBaebbnrfifHhDYfgasaacH8akY=wiFfYdH8Gipec8Eeeu0xXdbba9frFj0=OqFfea0dXdd9vqai=hGuQ8kuc9pgc9s8qqaq=dirpe0xb9q8qiLsFr0=vr0=vr0dc8meaabaqaciaacaGaaeqabaqabeGadaaakeaacqWGvbqvdaqhaaWcbaGaemyAaKgabaGaemysaKeaaaaa@3082@. For simplicity, auxiliaries *x*_*i *_are not shown.

To compute each family member's likelihood contribution including potential interventions, figure 2 shows the conditional dependencies between all pre/post-intervention quantities [25]. This graph shows the flow of information from carrier status to pre-intervention disease to intervention to post-intervention disease and will determine which quantities that the contribution requires or can ignore. The graph does not show any quantities in the intervention decision that are obviously unrelated to carrier status, like desire for children. Such quantities can be ignored because they provide no information about carrier status. In the graph, *T*_*i*_, *x*_*test*_, *U*_*i*_, *H*_-*i *_are the four factors that point to choosing intervention *I*_*i*_. But since *U*_*i *_does not connect back to *γ*_*i*_, it provides no information on carrier status. Thus only *T*_*i*_, *x*_*test*_, and *H*_-*i *_affect a person's decision to have an intervention and could yield information about carrier status. The likelihood contributions in equation (6) clearly depend on *T*_*i *_and *x*_*test*_. In addition, the contributions also condition on *H*_-*i*_: *H*_-*i *_only disappears from (6) because of assumption (2). The expressions below will explicitly show the conditioning on *H*_-*i *_to clarify that *H*_-*i *_is accounted for by the likelihood contributions. Thus the likelihood contributions contain all quantities related to both carrier status and intervention.

Each person's likelihood contribution depends on whether intervention was chosen. First consider the contribution from a person who did not choose intervention:

*P*(*I*_*i *_> *u*_*i*_, *H*_*i*_|*γ*_*i*_, *x*_*i*_, *H*_-*i*_) = *P*(*I*_*i *_> *u*_*i*_|*H*_*i*_, *γ*_*i*_, *x*_*i*_, *H*_-*i*_) × *P*(*H*_*i*_|*γ*_*i*_, *x*_*i*_).     (7)

Note that the second factor is the usual contribution from equation (6) that does not handle interventions. The first factor tries to extract information about carrier status from choosing not to undergo intervention. But by figure 2, as long as the full family history and any genetic test results are known, all three paths back to *γ*_*i *_are blocked. Thus there is no information about carrier status by choice of intervention once the full family history and genetic test results are known. Thus the first factor is independent of *γ*_*i *_and drops out of the likelihood. So the contribution if intervention was not chosen (7) is the same as of equation (6) that does not consider interventions:

*P*(*I*_*i *_> *u*_*i*_, *H*_*i*_|*γ*_*i*_, *x*_*i*_, *H*_-*i*_) ∝ *P*(*H*_*i*_|*γ*_*i*_, *x*_*i*_).

Next, the contribution from a person choosing intervention at age *I *is

*P*(*I*_*i *_= *I*, *H*_*i*_, HiI
 MathType@MTEF@5@5@+=feaafiart1ev1aaatCvAUfKttLearuWrP9MDH5MBPbIqV92AaeXatLxBI9gBaebbnrfifHhDYfgasaacH8akY=wiFfYdH8Gipec8Eeeu0xXdbba9frFj0=OqFfea0dXdd9vqai=hGuQ8kuc9pgc9s8qqaq=dirpe0xb9q8qiLsFr0=vr0=vr0dc8meaabaqaciaacaGaaeqabaqabeGadaaakeaacqWGibasdaqhaaWcbaGaemyAaKgabaGaemysaKeaaaaa@3068@|*γ*_*i*_, *x*_*i*_, *H*_-*i*_) = *P*(HiI
 MathType@MTEF@5@5@+=feaafiart1ev1aaatCvAUfKttLearuWrP9MDH5MBPbIqV92AaeXatLxBI9gBaebbnrfifHhDYfgasaacH8akY=wiFfYdH8Gipec8Eeeu0xXdbba9frFj0=OqFfea0dXdd9vqai=hGuQ8kuc9pgc9s8qqaq=dirpe0xb9q8qiLsFr0=vr0=vr0dc8meaabaqaciaacaGaaeqabaqabeGadaaakeaacqWGibasdaqhaaWcbaGaemyAaKgabaGaemysaKeaaaaa@3068@|*I*_*i *_= *I*, *H*_*i*_, *γ*_*i*_, *x*_*i*_) × *P*(*I*_*i *_= *I*|*H*_*i*_, *γ*_*i*_, *x*_*i*_, *H*_-*i*_) × *P*(*H*_*i*_|*γ*_*i*_, *x*_*i*_).

The last two factors can be treated the same as in equation (7), so the contribution is

∝ *P*(HiI
 MathType@MTEF@5@5@+=feaafiart1ev1aaatCvAUfKttLearuWrP9MDH5MBPbIqV92AaeXatLxBI9gBaebbnrfifHhDYfgasaacH8akY=wiFfYdH8Gipec8Eeeu0xXdbba9frFj0=OqFfea0dXdd9vqai=hGuQ8kuc9pgc9s8qqaq=dirpe0xb9q8qiLsFr0=vr0=vr0dc8meaabaqaciaacaGaaeqabaqabeGadaaakeaacqWGibasdaqhaaWcbaGaemyAaKgabaGaemysaKeaaaaa@3068@|*I*_*i *_= *I*, *H*_*i*_, *γ*_*i*_, *x*_*i*_)*P*(*H*_*i*_|*γ*_*i*_, *x*_*i*_).     (8)

The second factor is the pre-intervention contribution, and the first factor is an analogous post-intervention factor.

The post-intervention factor in (8) can be estimated from survival data. By figure 2, conditioning on *I*_*i *_(as the post-intervention factor does) breaks all links from UiI
 MathType@MTEF@5@5@+=feaafiart1ev1aaatCvAUfKttLearuWrP9MDH5MBPbIqV92AaeXatLxBI9gBaebbnrfifHhDYfgasaacH8akY=wiFfYdH8Gipec8Eeeu0xXdbba9frFj0=OqFfea0dXdd9vqai=hGuQ8kuc9pgc9s8qqaq=dirpe0xb9q8qiLsFr0=vr0=vr0dc8meaabaqaciaacaGaaeqabaqabeGadaaakeaacqWGvbqvdaqhaaWcbaGaemyAaKgabaGaemysaKeaaaaa@3082@ to both TiI
 MathType@MTEF@5@5@+=feaafiart1ev1aaatCvAUfKttLearuWrP9MDH5MBPbIqV92AaeXatLxBI9gBaebbnrfifHhDYfgasaacH8akY=wiFfYdH8Gipec8Eeeu0xXdbba9frFj0=OqFfea0dXdd9vqai=hGuQ8kuc9pgc9s8qqaq=dirpe0xb9q8qiLsFr0=vr0=vr0dc8meaabaqaciaacaGaaeqabaqabeGadaaakeaacqWGubavdaqhaaWcbaGaemyAaKgabaGaemysaKeaaaaa@3080@ and *γ*_*i*_. Thus UiI
 MathType@MTEF@5@5@+=feaafiart1ev1aaatCvAUfKttLearuWrP9MDH5MBPbIqV92AaeXatLxBI9gBaebbnrfifHhDYfgasaacH8akY=wiFfYdH8Gipec8Eeeu0xXdbba9frFj0=OqFfea0dXdd9vqai=hGuQ8kuc9pgc9s8qqaq=dirpe0xb9q8qiLsFr0=vr0=vr0dc8meaabaqaciaacaGaaeqabaqabeGadaaakeaacqWGvbqvdaqhaaWcbaGaemyAaKgabaGaemysaKeaaaaa@3082@ is independent non-informative censoring given *I*_*i*_, so standard survival analysis can estimate the post-intervention disease hazards λkI
 MathType@MTEF@5@5@+=feaafiart1ev1aaatCvAUfKttLearuWrP9MDH5MBPbIqV92AaeXatLxBI9gBaebbnrfifHhDYfgasaacH8akY=wiFfYdH8Gipec8Eeeu0xXdbba9frFj0=OqFfea0dXdd9vqai=hGuQ8kuc9pgc9s8qqaq=dirpe0xb9q8qiLsFr0=vr0=vr0dc8meaabaqaciaacaGaaeqabaqabeGadaaakeaaiiGacqWF7oaBdaqhaaWcbaGaem4AaSgabaGaemysaKeaaaaa@310E@(*y*|*I*_*i*_, *H*_*i*_, *γ*_*i*_). A simple way to do this is to fit a Cox model for time to disease with auxiliaries, pre-intervention disease history, and intervention age as time-dependent covariates [26]. The hazard ratios from this Cox model are multiplied with a pre-intervention hazard estimate (perhaps from the same dataset, or taken from other penetrance studies) to yield the post-intervention hazard. Then the post-intervention disease-specific survival is

SkI(y|γ)=exp{−∫IyλkI(u|Ii,Hi,γ) du}.     (9)
 MathType@MTEF@5@5@+=feaafiart1ev1aaatCvAUfKttLearuWrP9MDH5MBPbIqV92AaeXatLxBI9gBaebbnrfifHhDYfgasaacH8akY=wiFfYdH8Gipec8Eeeu0xXdbba9frFj0=OqFfea0dXdd9vqai=hGuQ8kuc9pgc9s8qqaq=dirpe0xb9q8qiLsFr0=vr0=vr0dc8meaabaqaciaacaGaaeqabaqabeGadaaakeaacqWGtbWudaqhaaWcbaGaem4AaSgabaGaemysaKeaaOGaeiikaGIaemyEaKNaeiiFaWhcciGae83SdCMaeiykaKIaeyypa0dcbiGae4xzauMae4hEaGNae4hCaa3aaiWabeaacqGHsisldaWdXaqaaiab=T7aSnaaDaaaleaacqWGRbWAaeaacqWGjbqsaaaabaGaemysaKeabaGaemyEaKhaniabgUIiYdGccqGGOaakcqWG1bqDcqGG8baFcqWGjbqsdaWgaaWcbaGaemyAaKgabeaakiabcYcaSiabdIeainaaBaaaleaacqWGPbqAaeqaaOGaeiilaWIae83SdCMaeiykaKIaeeiiaaIaemizaqMaemyDauhacaGL7bGaayzFaaGaeiOla4IaaCzcaiaaxMaadaqadaqaaiabiMda5aGaayjkaiaawMcaaaaa@5DEA@

Note that the hazards are cumulated starting from intervention age *I*. The post-intervention disease-specific density is fkI=λkISkI
 MathType@MTEF@5@5@+=feaafiart1ev1aaatCvAUfKttLearuWrP9MDH5MBPbIqV92AaeXatLxBI9gBaebbnrfifHhDYfgasaacH8akY=wiFfYdH8Gipec8Eeeu0xXdbba9frFj0=OqFfea0dXdd9vqai=hGuQ8kuc9pgc9s8qqaq=dirpe0xb9q8qiLsFr0=vr0=vr0dc8meaabaqaciaacaGaaeqabaqabeGadaaakeaacqWGMbGzdaqhaaWcbaGaem4AaSgabaGaemysaKeaaOGaeyypa0dcciGae83UdW2aa0baaSqaaiabdUgaRbqaaiabdMeajbaakiabdofatnaaDaaaleaacqWGRbWAaeaacqWGjbqsaaaaaa@39FA@. The likelihood contribution for a person who chose intervention is

P(Hi|γi)∝∏k=1DfkI(yikI|γi)cikISkI(uiI|γi)1−cikI×fk(yik|γi)cikSk(ui|γi)1−cik.     (10)
 MathType@MTEF@5@5@+=feaafiart1ev1aaatCvAUfKttLearuWrP9MDH5MBPbIqV92AaeXatLxBI9gBaebbnrfifHhDYfgasaacH8akY=wiFfYdH8Gipec8Eeeu0xXdbba9frFj0=OqFfea0dXdd9vqai=hGuQ8kuc9pgc9s8qqaq=dirpe0xb9q8qiLsFr0=vr0=vr0dc8meaabaqaciaacaGaaeqabaqabeGadaaakeaacqWGqbaucqGGOaakcqWGibasdaWgaaWcbaGaemyAaKgabeaakiabcYha8HGaciab=n7aNnaaBaaaleaacqWGPbqAaeqaaOGaeiykaKIaeyyhIu7aaebCaeaacqWGMbGzdaqhaaWcbaGaem4AaSgabaGaemysaKeaaaqaaiabdUgaRjabg2da9iabigdaXaqaaiabdseaebqdcqGHpis1aOGaeiikaGIaemyEaK3aa0baaSqaaiabdMgaPjabdUgaRbqaaiabdMeajbaakiabcYha8jab=n7aNnaaBaaaleaacqWGPbqAaeqaaOGaeiykaKYaaWbaaSqabeaacqWGJbWydaqhaaadbaGaemyAaKMaem4AaSgabaGaemysaKeaaaaakiabdofatnaaDaaaleaacqWGRbWAaeaacqWGjbqsaaGccqGGOaakcqWG1bqDdaqhaaWcbaGaemyAaKgabaGaemysaKeaaOGaeiiFaWNae83SdC2aaSbaaSqaaiabdMgaPbqabaGccqGGPaqkdaahaaWcbeqaaiabigdaXiabgkHiTiabdogaJnaaDaaameaacqWGPbqAcqWGRbWAaeaacqWGjbqsaaaaaOGaey41aqRaemOzay2aaSbaaSqaaiabdUgaRbqabaGccqGGOaakcqWG5bqEdaWgaaWcbaGaemyAaKMaem4AaSgabeaakiabcYha8jab=n7aNnaaBaaaleaacqWGPbqAaeqaaOGaeiykaKYaaWbaaSqabeaacqWGJbWydaWgaaadbaGaemyAaKMaem4AaSgabeaaaaGccqWGtbWudaWgaaWcbaGaem4AaSgabeaakiabcIcaOiabdwha1naaBaaaleaacqWGPbqAaeqaaOGaeiiFaWNae83SdC2aaSbaaSqaaiabdMgaPbqabaGccqGGPaqkdaahaaWcbeqaaiabigdaXiabgkHiTiabdogaJnaaBaaameaacqWGPbqAcqWGRbWAaeqaaaaakiabc6caUiaaxMaacaWLjaWaaeWaaeaacqaIXaqmcqaIWaamaiaawIcacaGLPaaaaaa@9686@

The contributions to the Bayes Factor in equation (1) are the ratio of likelihood contributions (10) for *γ*_*i *_= 1 to *γ*_*i *_= 0. [20] The post-intervention part of this ratio is

fkI(yikI|γi=1)cikISkI(uiI|γi=1)1−cikIfkI(yikI|γi=0)cikISkI(uiI|γi=0)1−cikI.     (11)
 MathType@MTEF@5@5@+=feaafiart1ev1aaatCvAUfKttLearuWrP9MDH5MBPbIqV92AaeXatLxBI9gBaebbnrfifHhDYfgasaacH8akY=wiFfYdH8Gipec8Eeeu0xXdbba9frFj0=OqFfea0dXdd9vqai=hGuQ8kuc9pgc9s8qqaq=dirpe0xb9q8qiLsFr0=vr0=vr0dc8meaabaqaciaacaGaaeqabaqabeGadaaakeaadaWcaaqaaiabdAgaMnaaDaaaleaacqWGRbWAaeaacqWGjbqsaaGccqGGOaakcqWG5bqEdaqhaaWcbaGaemyAaKMaem4AaSgabaGaemysaKeaaOGaeiiFaWhcciGae83SdC2aaSbaaSqaaiabdMgaPbqabaGccqGH9aqpcqaIXaqmcqGGPaqkdaahaaWcbeqaaiabdogaJnaaDaaameaacqWGPbqAcqWGRbWAaeaacqWGjbqsaaaaaOGaem4uam1aa0baaSqaaiabdUgaRbqaaiabdMeajbaakiabcIcaOiabdwha1naaDaaaleaacqWGPbqAaeaacqWGjbqsaaGccqGG8baFcqWFZoWzdaWgaaWcbaGaemyAaKgabeaakiabg2da9iabigdaXiabcMcaPmaaCaaaleqabaGaeGymaeJaeyOeI0Iaem4yam2aa0baaWqaaiabdMgaPjabdUgaRbqaaiabdMeajbaaaaaakeaacqWGMbGzdaqhaaWcbaGaem4AaSgabaGaemysaKeaaOGaeiikaGIaemyEaK3aa0baaSqaaiabdMgaPjabdUgaRbqaaiabdMeajbaakiabcYha8jabeo7aNnaaBaaaleaacqWGPbqAaeqaaOGaeyypa0JaeGimaaJaeiykaKYaaWbaaSqabeaacqWGJbWydaqhaaadbaGaemyAaKMaem4AaSgabaGaemysaKeaaaaakiabdofatnaaDaaaleaacqWGRbWAaeaacqWGjbqsaaGccqGGOaakcqWG1bqDdaqhaaWcbaGaemyAaKgabaGaemysaKeaaOGaeiiFaWNaeq4SdC2aaSbaaSqaaiabdMgaPbqabaGccqGH9aqpcqaIWaamcqGGPaqkdaahaaWcbeqaaiabigdaXiabgkHiTiabdogaJnaaDaaameaacqWGPbqAcqWGRbWAaeaacqWGjbqsaaaaaaaakiabc6caUiaaxMaacaWLjaWaaeWaaeaacqaIXaqmcqaIXaqmaiaawIcacaGLPaaaaaa@90C3@

Note that if the hazard ratios between carriers and non-carriers are equal, then at the age of oophorectomy itself, the densities for carriers and non-carriers are equal to each other (and same for the survivals), and thus (11) is one. At ages beyond the age of oophorectomy, the hazards start cumulating as in equation (9), and the densities and survivals will begin to differ and (11) will differ from one. The amount of information in oophorectomy can be measured by the ratio of hazard ratios of carriers to non-carriers; the further this ratio is from one, the further (11) is one will be from one, and thus the more important it is to account for oophorectomy. However, a hazard ratio of one still has implications for all post-oophorectomy ages, so we stress that ratio of one still must be accounted for.

### Incorporate Oophorectomy into BRCAPRO

Including an intervention requires estimating post-intervention disease-specific hazards, the simplest way being multiplying each pre-intervention hazard by a hazard ratio to get post-intervention hazards. BRCAPRO uses pre-oophorectomy hazards estimated by [27]. We use the most recently estimated hazard ratios for obtaining post-oophorectomy breast cancer hazards for mutation carriers [15]. It is critical to consider all factors that could modify the appropriate hazard ratio to use. For example, [15] estimates hazard ratios for breast cancer within groups defined by pre-oophorectomy disease history, age at oophorectomy, time since oophorectomy, and by BRCA1 vs. BRCA2. Although [15] does not formally test if the hazard ratios differ within each group, we must informally assess whether the differences they found are strongly statistically significant. For example, [15] finds that those with BRCA1 mutations have a hazard ratio of 0.43 (0.29, 0.65) and those with BRCA2 mutations a hazard ratio of 0.57 (0.28, 1.15). Since these two estimates must be nearly independent, we can calculate a p-value of 0.50 for the difference in hazard ratios between the two loci; thus we are justified in using the same hazard ratio for both loci. For age at oophorectomy, we cannot calculate a p-value, but the degree of overlap of the confidence intervals between age ranges in [15] suggests that the differences in the hazard ratio are probably statistically insignificant, and thus justifies the use of a common hazard ratio over all ages. The overall hazard ratio found by [15] was 0.46 (0.32, 0.65), but for 15 years after oophorectomy, they find a hazard ratio of 1.30 (0.51, 3.30). The two intervals overlap, but it's possible that the two are significantly different. However, neither [16] nor [28] noticed this in their data, and the 1.30 hazard ratio is estimated quite imprecisely, making us hesitant to use this in a model for clinical decision-making. Also, if only a few modifying factors exist, using a single hazard ratio for everyone is advantageous because this overall hazard ratio would be most precisely estimated and is relevant for most consultands. Thus we use the overall hazard ratio of 0.46.

For oophorectomy and ovarian/peritoneal cancers among mutation carriers, we combined three papers: one retrospective study reports an overall hazard ratio of 0.04 with CI (0.01,0.16) [16] and two prospective studies report 0.15 with CI (0.02,1.31) [28] and 0.20 with CI (0.07,0.58) [11]. Since all references combine ovarian cancer and primary peritoneal carcinoma into a single endpoint, separate effects of oophorectomy on each cancer cannot be estimated, so we combine them into a single endpoint. All papers report that these hazard ratios do not depend on pre-oophorectomy disease history, age at oophorectomy, time since oophorectomy, or by *BRCA1 *vs. *BRCA2*. We combine these three results with a fixed-effect meta-analysis [29] to average the hazard ratios weighted by their inverse variances, yielding an estimate of 0.12 with CI (0.05,0.25).

Unfortunately, there are no comparable studies of oophorectomy in *BRCA *non-carriers, only studies that mix carriers with non-carriers. A population-based study found a hazard ratio of 0.50 [30] and another study among women with family history of breast cancer reported a hazard ratio of 0.41 [31]. Since these ratios are close to the carrier ratio, we set non-carriers to have the same hazard ratio of 0.46 as the carriers. For ovarian/peritoneal cancer, the only comparable study among non-carriers reports a hazard ratio of 0.05 with CI (0.01,0.22) [19]. Although [19] doesn't report a p-value testing for different effects of oophorectomy in carriers vs. non-carriers, since their two estimates should be nearly independent, we calculate a p = 0.048 that the two effects are different. Thus we set the non-carrier hazard ratio for oophorectomy to 0.05.

## Results

### Including Oophorectomy into BRCAPRO

This section computes the post-intervention contributions to the BRCAPRO Bayes Factor (as in equation (11)) from a woman who underwent oophorectomy at age 35, for all three possibilities of developing breast or peritoneal cancer, or current age without either cancer (remember that ovarian cancer is eliminated by oophorectomy, but the closely-related peritoneal cancer can still occur).

**Figure 3:**
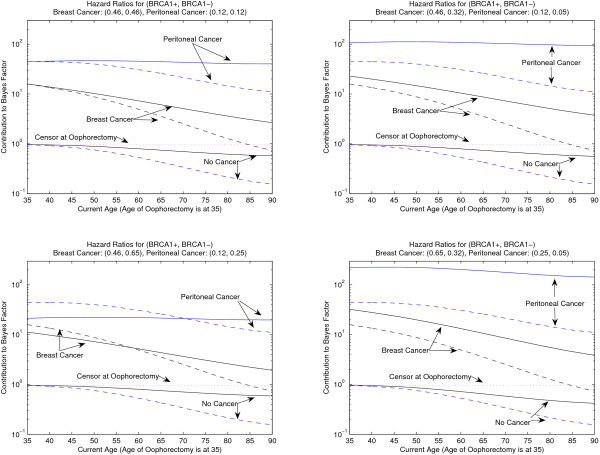
**Effect of ignoring and accounting for oophorectomy on BRCAPRO**. Effect of ignoring and accounting for an oophorectomy at age 35 on a relative's post-oophorectomy contribution to the BRCAPRO Bayes Factor for *BRCA1*. Solid lines account for the oophorectomy, but dashed lines ignore the oophorectomy, and the dotted line censors the relative at their age of oophorectomy. The person got peritoneal cancer, breast cancer, or no cancer at the age indicated on the x-axis. The four panels vary the hazard reduction due to oophorectomy. In the top left, the hazard reduction due to oophorectomy is the same for *BRCA1 *carriers and non-carriers: 0.46 and 0.12, on time to subsequent breast and peritoneal cancer respectively. In the top right, the hazard reduction for oophorectomy in *BRCA1 *non-carriers increases to 0.32 and 0.05 for breast and peritoneal cancer respectively. In the bottom left, the hazard reduction for oophorectomy in *BRCA1 *carriers decreases to 0.65 and 0.25 for breast and peritoneal cancer respectively. The bottom right has hazard reductions for breast cancer of 0.65 and 0.32 in carriers and non-carriers respectively and hazard reductions for peritoneal cancer of 0.25 and 0.05 in carriers and non-carriers respectively.

Figure 3 plots a woman's post-oophorectomy contribution to the *BRCA1 *Bayes Factor, by accounting for her oophorectomy at age 35 (solid lines), ignoring her oophorectomy (dashed lines), or censoring her at the age of oophorectomy (dotted lines). A woman can get breast or peritoneal cancer, or no cancer, at the age indicated on the x-axis. Since the effect of oophorectomy in non-carriers has not been well-studied, each panel of figure 3 considers four different possible effects of oophorectomy in non-carriers: the top left has the equal hazard reductions in carriers and non-carriers, the top right has more hazard reduction in non-carriers, the bottom left has more hazard reduction in carriers, and bottom right has extreme (but reasonable) difference in hazard reduction between carriers and non-carriers. The dashed and dotted lines are the same in all figures since they never account for the effect of oophorectomy.

The top left panel of figure 3 uses the equal hazard ratios (see Methods) of 0.46 for breast cancer and 0.12 for peritoneal cancer for carrier and non-carriers. Equal hazard ratios means that carriers and non-carriers benefit the same from oophorectomy. Ignoring oophorectomy leads to underestimated contributions in all cases, and thus the carrier probability will be underestimated, as for the family in figure 1. At at age 35, the solid and dashed lines are the same because the hazard ratios in carriers and non-carriers are the same. The solid and dashed lines diverge at older ages, as the woman lives more of her life without ovaries, and by age 90 there is roughly a factor of five change in the contribution. For a woman who did not get cancer, in this scenario, it is better to censor her at her age of oophorectomy than at the latest age known without cancer.

In the top right panel of figure 3, the non-carrier hazard ratios decrease to 0.32 and 0.05 (the lower end of the CIs from Methods) for breast and peritoneal cancer respectively. In this scenario, oophorectomy benefits non-carriers more than carriers and thus developing cancer after oophorectomy is additional evidence for being a carrier. Indeed, in this panel, there is more difference between the solid and dashed lines than in the top left panel. Furthermore, not developing cancer after oophorectomy is usually less evidence for being a non-carrier since oophorectomy naturally lowers cancer risk for everyone. The bottom left panel of figure 3 increases the non-carrier hazard ratios to 0.65 and 0.25 (the upper end of the CIs from Methods) for breast and peritoneal cancer respectively. In this scenario, carriers benefit more from oophorectomy. Now cancer after oophorectomy is less evidence of being a carrier, so the solid lines accounting for oophorectomy decrease and cross over the dashed lines ignoring oophorectomy. In this case, ignoring oophorectomy could lead to either under- or over-estimation of the BF contributions and carrier probability, depending on the ages of the cancers after oophorectomy.

The bottom right panel of figure 3 shows an extreme, but reasonable, case where oophorectomy is most informative about carrier status. The benefit to non-carriers is set to 0.32 and 0.05 (the lower end of the CIs from Methods) and for carriers is set to 0.65 and 0.25 (the upper end of the CIs from Methods) for breast and peritoneal cancer respectively. These non-carrier benefits are as different as possible from the carrier benefits, but plausible since they are within the CIs. Using these benefits, for breast cancer by age 90, there is a factor of 8 underestimation of the contributions if oophorectomy is ignored from someone with breast cancer, and 15 if peritoneal cancer. Although the difference between hazard ratios of 0.25 versus 0.05 seems small, their ratio is large, and it is the ratio that matters. If we used the hazard ratios in the top right panel to estimate the carrier probability for the family in figure 1, the carrier probability increases to 12%. Furthermore, if the mother in figure 1 got peritoneal cancer at age 80 instead of breast cancer, the carrier probability ignoring her oophorectomy is 25%, the probability goes to 31% under equal hazard ratios of 0.12, the probability goes to 39% assuming hazard ratios of 0.12 and 0.05 for carriers and non-carriers (as in the top left panel and as set in BRCAPRO), and the probability goes to 43% for the hazard ratios in the bottom right panel.

## Discussion

People with a family history of disease may undergo prophylactic interventions to prevent future disease. Interventions are informative about the carrier probability not only because interventions reduce the penetrance of mutations, but also because only those with high disease risk (and thus potential mutation carriers) will opt for it. Furthermore, as interventions become more commonly undergone (most studies show that about 50% of *BRCA *carriers undergo oophorectomy [12]) and consultands report more family members as having undergone intervention, it is increasingly important that mutation prediction models account for interventions. Extending Mendelian models to reflect the latest research findings helps answer the call to translate genetic research for use by genetic counselors [3].

Incorporating interventions into Mendelian mutation prediction models requires only a post-intervention factor multiplying the likelihood contribution from family members with the intervention. The only new quantities required are the post-intervention disease hazards which can be estimated by multiplying the pre-intervention hazards by hazard ratios for the effect of intervention that can be found in published studies of intervention effects. We extended the BayesMendel software to handle interventions, and the new BRCAPRO that accounts for oophorectomy is available for clinical use. Other interventions that BayesMendel could incorporate are prophylactic tamoxifen therapy into BRCAPRO, and colectomy and hysterectomy with oophorectomy into MMRPRO.

To incorporate oophorectomy into BRCAPRO, we relied on hazard ratios for the effect of oophorectomy in *BRCA *mutation carriers found in previous studies [11,16,28], and for non-carriers and peritoneal cancer [19]. Although no comparable studies exist for breast cancer hazard ratios in non-carriers, existing studies that likely involve predominantly non-carrier subjects [30,31] show hazard ratios similar to those in carriers, so we set hazard ratios in non-carriers equal to that of carriers. However, it is more likely that *BRCA *mutation carriers benefit less from oophorectomy because they have earlier age of onset of breast cancer. Since our incorporation sets equal benefits to prevent breast cancer, this may underestimate the impact of oophorectomy, but further studies of oophorectomy in non-carriers are needed to clarify this situation. Fortunately, forthcoming prospective studies of the benefit of oophorectomy in women at high risk of ovarian cancer, such as GOG-0199 [32], could provide the data required to refine the incorporation of oophorectomy into BRCAPRO. Such studies will include *BRCA *mutation non-carriers, allowing estimation of the intervention hazard ratio in non-carriers.

Figure 3 shows the effect of incorporating oophorectomy for different possible hazard ratios. All four panels of figure 3 show that accounting for oophorectomy is most important for older family members with oophorectomy. Ignoring oophorectomy leads to underestimated carrier probabilities, unless carriers benefit more from oophorectomy, as in the lower left panel of figure 3. The amount of underestimation depends on how informative post-oophorectomy diseases are for carrier status. The top left panel of figure 3 is for equal carrier and non-carrier hazard ratios, and the effect of incorporating oophorectomy here is not as great as in the two right panels, where carriers benefit less from oophorectomy. Interventions are most informative about carrier status when carriers benefit less than non-carriers, as measured by the ratio of the hazard ratios in carriers to non-carriers.

Our incorporation of interventions shows that there is no information about carrier status in whether a relative chooses intervention or not, as long as the full family history and genetic test results are known. This is reflected in the fact that the first factor of equation (7) is ignorable. This factor will not be ignorable if the relative based her intervention decision on factors predictive of carrier status that are not available to the consultand. For example, if a consultand is unaware of genetic test results on a relative who opted for or against intervention based on those test results, then the first factor is not ignorable. However, the first factor can be estimated from a survival analysis of age at intervention given pre-intervention disease history and carrier status. Prospective studies such as GOG-0199 could provide the required data.

Although this paper has emphasized using Mendelian models in genetic counseling, other applications exist. Mendelian models can help gene characterization research by helping to select high-risk individuals for studies (such as in GOG-0199) and also to help build statistical models that estimate individualized disease risks [7]. Both applications benefit from the incorporation of interventions into Mendelian models.

## Conclusion

These results show that not accounting for medical interventions can lead to seriously misleading carrier probability estimates that could affect a clinician's recommendation about offering genetic testing. This is especially true if an intervention was undergone long ago by a family member or if the intervention has different effects on carriers versus non-carriers. The new BayesMendel software has been extended to allow medical interventions, so any carrier probability model can easily account for interventions.

## Competing interests

The author(s) declare that they have no competing interests.

## Pre-publication history

The pre-publication history for this paper can be accessed here:


